# Bone Marrow Aspirate Concentrate-Enhanced Marrow Stimulation of Chondral Defects

**DOI:** 10.1155/2017/1609685

**Published:** 2017-05-14

**Authors:** Henning Madry, Liang Gao, Hermann Eichler, Patrick Orth, Magali Cucchiarini

**Affiliations:** ^1^Institute of Experimental Orthopaedics and Osteoarthritis Research, Saarland University, Kirrberger Strasse, Building 37, 66421 Homburg, Saar, Germany; ^2^Department of Orthopaedic Surgery, Saarland University Medical Center, Kirrberger Strasse, Building 37, 66421 Homburg, Saar, Germany; ^3^Institute for Clinical Haemostaseology and Transfusion Medicine, Saarland University, Kirrberger Strasse, Building 1/Building 57, 66421 Homburg, Saar, Germany

## Abstract

Mesenchymal stem cells (MSCs) from bone marrow play a critical role in osteochondral repair. A bone marrow clot forms within the cartilage defect either as a result of marrow stimulation or during the course of the spontaneous repair of osteochondral defects. Mobilized pluripotent MSCs from the subchondral bone migrate into the defect filled with the clot, differentiate into chondrocytes and osteoblasts, and form a repair tissue over time. The additional application of a bone marrow aspirate (BMA) to the procedure of marrow stimulation is thought to enhance cartilage repair as it may provide both an additional cell population capable of chondrogenesis and a source of growth factors stimulating cartilage repair. Moreover, the BMA clot provides a three-dimensional environment, possibly further supporting chondrogenesis and protecting the subchondral bone from structural alterations. The purpose of this review is to bridge the gap in our understanding between the basic science knowledge on MSCs and BMA and the clinical and technical aspects of marrow stimulation-based cartilage repair by examining available data on the role and mechanisms of MSCs and BMA in osteochondral repair. Implications of findings from both translational and clinical studies using BMA concentrate-enhanced marrow stimulation are discussed.

## 1. Introduction

Mesenchymal stem cells (MSCs) play a key role in articular cartilage repair. MSCs have multilineage differentiation potential, allowing them to differentiate, for example, into chondrocytes and osteoblasts, the key cells from the two tissues that constitute the osteochondral unit. They were first isolated from the bone marrow, and the potency of MSCs is currently being employed in the techniques of marrow stimulation for symptomatic small chondral defects. If bone marrow fills a cartilage defect either as a result of marrow stimulation for chondral defects or the course of the spontaneous repair of osteochondral defects, a bone marrow clot forms within the cartilage defect. Pluripotent MSCs from the subchondral bone marrow are subsequently mobilized, migrate into the defect filled with the clot, and differentiate into chondrocytes and osteoblasts. Over time, they form a fibrocartilaginous repair tissue in the defect and close the connection with the subchondral bone.

Bone marrow stem cells have been successfully transformed into several cell types among which chondrocytes, osteoblasts, adipocytes, angioblasts [1], and neural cells [2], to potentially be used to treat a variety of illnesses [3–6]. In the orthopaedic field, additional application of a bone marrow aspirate (BMA) to the procedure of marrow stimulation has been recently studied, since the bone marrow itself is both a source of MSCs, providing a cell population capable of chondrogenesis and of various growth factors stimulating cartilage repair [7–10]. Moreover, the bone marrow clot provides a three-dimensional (3D) environment which supports the chondrogenesis of MSCs. Finally, it is possible that it protects the subchondral bone plate and the subarticular spongiosa from structural alterations of its microarchitecture.

In contrast to the cost- and labor-intensive cultivation and propagation of cells such as MSCs or articular chondrocytes, the clinical use of “minimally processed” autologous BMA that can be prepared in the operation room as a single-step procedure appears straightforward. Native and concentrated BMA have been intensively studied in the context of articular cartilage repair. Such enhanced techniques of marrow stimulation have been shown to improve articular cartilage repair in both animal models and patients.

The purpose of this review is to bridge the gap in our understanding between the basic science knowledge about MSCs and BMA on one side and the clinical and technical aspects of marrow stimulation-based cartilage repair on the other side by examining available data on the role and mechanisms of MSCs and BMA in osteochondral repair. A focus is on the steps of mobilization of cells from the subchondral bone and repair tissue formation, including adherence of the bone marrow clot to the subchondral bone. The implications of findings from both translational and clinical studies using BMA concentrate-enhanced marrow stimulation are also discussed.

## 2. Marrow Stimulation-Based Cartilage Repair

**Figure 1:**
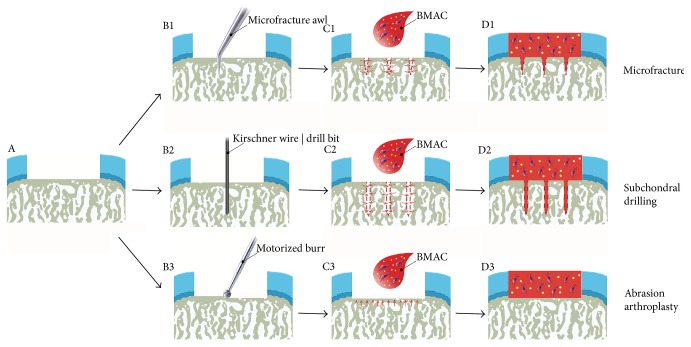
Principle of bone marrow aspirate concentrate- (BMAC-) enhanced marrow stimulation. (a) Schematic view of a full-thickness focal chondral defect. (b) Marrow stimulation can be performed with microfracture (b1), subchondral drilling (b2), or abrasion arthroplasty (b3). The subchondral bone plate can be perforated with a microfracture awl (microfracture), a Kirschner wire or a drill bit (subchondral drilling), or a motorized burr (abrasion arthroplasty) (c). After marrow stimulation, bone marrow containing mesenchymal stem cells ascends from the marrow cavity of the underlying subchondral bone via the channels generated by the marrow stimulation procedures. The defects are filled with a clot of autologous BMAC, containing mesenchymal stem cells and growth factors which possibly favor new tissue formation. (d) Defects thus contain bone marrow both from the subchondral bone and the additional BMAC application, and gradually a cartilaginous repair tissue forms within them. Red dashed lines (c1, d1, c2, and d2) show the outline of holes created by microfracture and subchondral drilling. Red arrows (c1, c2, and c3) within the subchondral bone denote the migration direction of the liquid bone marrow.

Marrow stimulation techniques are the most important first-line treatment options for small symptomatic articular cartilage defects [11]. Their principle is to establish a communication of the cartilage defect with the subchondral bone marrow compartment (Figure 1). First, the cartilage defect is surgically prepared in a meticulous fashion, including removal of cartilage fragments and generation of stable and vertically oriented margins of the peripheral cartilage. The next step is the preparation of the bony defect base. Here, the entire calcified cartilage layer has to be removed, thereby exposing the superficial part of the subchondral bone plate without damaging it.

Marrow stimulation is performed by one of three different techniques. Microfracture induces multiple holes of the subchondral bone plate [12]. These focal perforations are the result of forcing the sharp tip of a microfracture awl into the subchondral bone plate. The impaction of the conical or polyhedral awl tip induces multiple small injuries of the adjacent bone. Subchondral drilling, proposed already in 1957 [13], is often termed Pridie drilling [14]. Here, the tip of a small diameter bone cutting device such as a drill bit or a Kirschner wire (K-wire) is placed on the base of the prepared cartilage defect and, at high speed, the rotating instrument cuts through the subchondral bone plate into the subarticular spongiosa. A defined number of standardized cylindrical holes are the result of subchondral drilling. Abrasion arthroplasty, in contrast, refers to a generalized abrasion of the subchondral bone plate of limited depth [15]. The small bony canals within the subchondral bone plate are opened following an abrasion with a round burr by removing about 1.0–1.5 mm of its thickness without completely eliminating the subchondral bone plate. This exposes the vascularity of the subchondral bone plate, providing the connecting link to the subchondral bone marrow. Although it has been suggested that larger holes may allow for an amplified access of reparative elements from the subchondral bone [5], recent data from translational models support the use of small diameter devices, most likely due to a lesser structural disturbance of the microarchitecture of the subchondral bone plate and subarticular spongiosa [16, 17].

After the communication of the cartilage defect with the subchondral bone marrow compartment has been established, bone marrow from the subchondral bone fills the defect, a clot forms, and more pluripotent progenitor cells from the subchondral compartment subsequently migrate into the defect, differentiate into chondrocytes, and, over time, form a fibrocartilaginous repair tissue. This repair tissue also serves to stabilize the adjacent cartilage and prevent early osteoarthritic degeneration.

## 3. Mesenchymal Stem Cells and Their Role in Osteochondral Repair

### 3.1. Definitions

MSCs represent a small fraction (0.001–0.01%) of nonhematopoietic, multipotent cells of the bone marrow. They are also present in other tissues including the synovium, periosteum, trabecular bone, adipose tissue, skeletal muscle, circulatory system, placenta, umbilical cord blood, and Wharton's jelly [18].

MSCs exhibit a potent ability for self-renewal, stemness, and commitment toward cells of the mesodermal lineage (cartilage, bone, fat, muscle, meniscus, and tendons/ligaments) [19, 20]. MSCs have been defined by the Mesenchymal and Tissue Stem Cell Committee of the International Society for Cellular Therapy via a minimal set of standard criteria as being plastic-adherent in standard culture conditions, expressing CD105, CD73, and CD90 at their surface while lacking CD45, CD34, CD14 (or CD11b), CD79*α* (or CD19), and HLA-DR, and being able to differentiate in chondrocytes, osteoblasts, and adipocytes in vitro [21].

MSCs have homing, reparative, and trophic properties, migrating in damaged tissues upon recruitment from the circulation from the perivascular niche via activation of adhesion molecules (integrins, chemokine receptors) upon the release of factors from injured cells (chemokines) [22]. Once mobilized, MSCs produce a number of factors that can impact the healing responses locally by reduction of cell apoptosis, fibrosis, and inflammation and by activation of cell proliferation, mobilization, differentiation, and angiogenesis via paracrine and autocrine pathways [23]. Key agents involved in these processes include the vascular endothelial growth factor (VEGF), hepatocyte growth factor (HGF), insulin-like growth factor I (IGF-I), basic fibroblast growth factor (FGF-2), transforming growth factor beta (TGF-*β*), granulocyte-macrophage colony-stimulating factor (GMCSF), monocyte chemotactic protein-1 (MCP-1), macrophage inflammatory proteins-1 (MIP-1*α*, MIP-1*β*), regulated upon activation, normal T-cell expressed and secreted (RANTES), growth-related genes (GRO*α*, GRO*β*), stromal-derived factor 1 (SDF-1), interleukin 6 (IL-6), angiopoietin-1, and stem cell factor (SCF). Such factors most likely coexist especially when MSCs are provided in a site of tissue injury like in a BMA. In such a setup, these agents may thus have the potential to cointeract in order to exert their specific activities via molecular interplays and subsequently to promote optimal MSC-associated therapeutic tissue healing in particular in a highly concentrated environment [24]. MSCs also have immunosuppressive activities, balancing or even inhibiting the activation of NK cells, dendritic cells, macrophages, and T and B lymphocytes [25]. In this regard, there is evidence that MSCs display advantageous anti-inflammatory and antifibrotic activities together with a potency for migration within a site of inflammation [26, 27], allowing to favorize their safe implantation and therapeutic effects in tissue lesions.

### 3.2. MSCs in Osteochondral Repair

Bone marrow MSCs undergo both chondrogenic and osteogenic differentiation processes [28, 29] that may promote the healing of the entire, damaged osteochondral unit:

(1) Chondrogenic MSC differentiation and subsequent cartilage formation are characterized by the expression of cartilage-specific markers (sex-determining region Y SRY-box transcription factors 5, 6, and 9; SOX5, SOX6, and SOX9; aggrecan; biglycan; type II, type IX, and type XI collagen; decorin; cartilage oligomeric matrix protein; COMP; scleraxis).

(2) Osteogenic MSC differentiation and subsequent bone formation are noted upon expression of bone-specific markers (Runt-related transcription factor 2 (RUNX2); alkaline phosphatase (ALP); type I collagen; bone sialoprotein (BSP); osteopontin (OPN); osteocalcin (OCN); calcium-rich mineralized matrix).

## 4. Mobilization of Cells from the Subchondral Bone into Sites of Articular Cartilage Damage

Mobilization of cells from the subchondral bone into sites of articular cartilage damage is possible once a communication of the defect with the subchondral bone marrow has been established. Liquid bone marrow fills the defect, and a clot forms. Theoretically, a bone marrow clot completely filling a full-thickness chondral defect of 2 cm in diameter in the medial femoral condyle would have a volume of about 300 *μ*l (assuming a mean cartilage thickness at this topographical location of 2 mm) [30].

The cascade of bone marrow clotting is initiated by thrombocytes. They adhere to the exposed collagen of the subchondral bone with the aid of von Willebrand factor which creates a bridge between the platelets and the basis of the defect. Interestingly, platelets do not adhere to normal (uninjured) articular cartilage, and cartilage does not induce platelet aggregation in vivo, a discovery ascribed to the proteoglycans which block lysine sites on the collagen molecule [31]. If cartilage is treated with proteolytic enzymes such as trypsin, the tissue is rendered active as a platelet aggregant and adhesion and aggregation of platelets on the surface of the lesions results [31]. This highlights the importance of preparing the cartilage defect to be able to induce such platelet-subchondral bone/cartilage interactions. The aggregated platelets then degranulate, releasing several factors among which ILs and growth factors such as platelet-derived growth factor (PDGF), VEGF, and TGF-*β* which further potentiate their activation. PDGF, TGF-*β*, and other factors such as IGF-I also serve as chemoattractants. Next, the complement, kinin, plasminogen, and clotting cascades are activated. Fibrin is formed by polymerization through the clotting cascade, generating a mesh of long strands of insoluble protein that trap the cells, thus forming a clot in the cartilage defect that serves as a scaffold for adhesion. Little is known on the specific mechanisms on how cells are recruited to the site of the defect and how the cells move through the subchondral space. In accordance with wound healing, neutrophils and monocytes concentrate at the site of injury and direct the breakdown of the clot. The macrophages release chemoattractant substances and growth factors that recruit additional cells and stimulate collagen production. Over a period of several weeks, more pluripotent progenitor cells migrate through the openings of the subchondral bone into the articular cartilage defect. In the defect, they proliferate and undergo differentiation, together with matrix deposition and osteochondral remodeling.

From the classical paper of Shapiro et al. in a rabbit model [32], the time course of chondro- and osteogenesis in an osteochondral defect is well known. Fibrinous arcades are established in the first days across the defect, spanning both edges of the adjacent uninjured cartilage. Undifferentiated MSC ingrowth from the subchondral bone marrow can be seen after 7 days. The MSCs proliferate and differentiate into fibroblasts, articular chondroblasts, and osteoblasts. The first evidence of synthesis of a cartilaginous extracellular matrix, as defined by safranin-O staining, appears at about 10 days. The new repair tissue first contains flattened fibrocartilaginous cells and then round cells similar to chondrocytes. The defect is completely repopulated after about 6 weeks, with synthesis of repair cartilage and bone matrices in their appropriate locations. At 24 weeks, both the tidemark and the subchondral bone plate are re-established. The initially formed cancellous woven bone is replaced by the lamellar cancellous bone. Over time, the penetrations of the subchondral bone plate are closed with newly deposited bone. The cellular contribution to cartilage repair from the adjacent cartilage is very low [32].

During the repair of the osteochondral unit, osteogenesis is supported by the mobilization of perivascular cells and blood vessels within the subchondral bone compartment [33]. Under physiologic conditions, numerous arterial terminal branches and venous plexus are located in the subchondral bone plate [34, 35] and the blood flow rate is up to 10 times higher than in the cancellous bone, ensuring nutrient and water supply to the articular cartilage [35]. The physiological avascularity of the articular cartilage [36] is retained by the presence of antiangiogenic factors such as thrombospondin-1 [37, 38], thrombospondin-2 [39], troponin-I [40], tenascin [41], tissue inhibitors of metalloproteinases (TIMP) 1 and 2 [42], and chondromodulin-1 [37, 43–45]. While original hyaline articular cartilage is rich in these proteins, fibrocartilaginous repair tissue has been shown to lack such factors [37, 43], consequently permitting blood vessel invasion and stimulation of both endochondral and intramembranous bone formation [32]. Furthermore, in certain pathologies such as osteochondral defects [46], osteoarthritis [42, 47–49], and inflammatory joint diseases [50–53], chondrocytes have been demonstrated to express various proangiogenic factors, including VEGF [46, 54–56], connective tissue growth factor (CTGF) [57], FGF-2 [42], tumor necrosis factor-*α* (TNF-*α*) [58], and matrix metalloproteinases (MMP) 9 and 13 [59, 60]. Interestingly, blocking VEGF with its soluble antagonist (sFlt1) [61] or the antibody bevacizumab [62] improved the chondrogenic potential of stem cells [61] and articular cartilage repair in vivo [62]. Thus, although vascularization is beneficial for osteogenesis and subchondral bone reconstitution, it may critically disturb the repair of the overlying articular cartilage.

## 5. Bone Marrow Aspirate and Its Concentrate

### 5.1. Bone Marrow Aspirate

Bone marrow produces the red blood cells during hematopoiesis and is a major component of the immune system by producing lymphocytes. It mainly consists of hematopoietic tissue and fat cells. In the stroma of the bone marrow, supporting cells such as fibroblasts, macrophages, adipocytes, osteoblasts, osteoclasts, and endothelial cells are present. Myelopoietic and erythropoietic cells together with the lymphocytes are the major cell types. The bone marrow also contains hematopoietic cells and MSCs (also termed marrow stromal cells). Bone marrow aspiration can be performed with local anesthetic under guidance of ultrasound or fluoroscopic imaging to improve accuracy and efficiency.

### 5.2. Bone Marrow Aspirate Concentrate

Unprocessed BMA is been rarely used. As only about 0.001% of nucleated cells from BMA are MSCs [63], attempts are made to increase their number, usually by concentrating the autologous BMA by density-gradient centrifugation. This concept of concentrating BMA to produce BMA concentrate (BMAC) allows increasing not only the numbers of MSCs but also platelets containing growth factors and hematopoietic stem cells (HSCs) per sample volume. MSCs present strong self-renewal abilities with a differentiation capacity to form chondrocytes, osteocytes, and adipocytes. The platelet component of BMAC releases growth factors to initiate stem cell migration to the injury site and provides adhesion sites for the migrating stem cells [10]. Moreover, HSCs provide support to the vasculature system by differentiating into blood cells and maintain cell-to-cell contact with MSCs, stimulating osteogenesis.

## 6. Bone Marrow Aspirate-Enhanced Marrow Stimulation

BMA-enhanced marrow stimulation is based on a prior treatment of the subchondral bone plate in the defects with marrow stimulation, although BMA has been also applied in few cases to cartilage defects that were only debrided down to the subchondral bone. Microfracture is the main marrow stimulation technique performed, providing a more roughened base of the defect caused by the several microfracture holes and possible minute fractures. By definition, the continuous bleeding from the microfracture holes may always contribute to the final composition of the bone marrow clot. As the stability of the clot has been highlighted since a long time, alternative methods seek to enhance the security of the bone marrow clot. These include the application of bioresorbable membranes, thought to provide an additional 3D environment for the cells undergoing chondrogenesis in the cartilage defect.

## 7. Effects of Bone Marrow Aspirate-Enhanced Marrow Stimulation on Articular Cartilage Repair

### 7.1. Translational Evidence of BMAC-Enhanced Marrow Stimulation

The effect of unconcentrated BMA to enhance osteochondral repair has, to the best of our knowledge, currently not been reported to date in either translational or clinical settings. Only two preclinical studies applied marrow stimulation enhanced with BMAC (termed BMAC-enhanced marrow stimulation) to analyze the repair of chondral lesions (Table 1). Both studies were performed in the knee joint of large animal models (goat [64] and horse [65], respectively). Despite the availability of data regarding BMAC-enhanced repair of osteochondral lesions by the use of gene vectors [7, 66] or scaffolds [67–70], the particular effect of BMAC-enhanced marrow stimulation for chondral lesions has not yet been evaluated in other joints or experimental animals. Such scaffolds may aid to provide a stable environment for the clot and subsequent repair tissue development. However, little is known about the mechanisms of their interaction with the BMAC-enhanced marrow stimulation process.

Fortier et al. treated 12 young adult horses with full-thickness chondral defects (15 mm in diameter, 3 mm in depth) in the trochlear ridge with microfracture alone (6 microfracture holes per defect) or BMAC-enhanced microfracture [65]. BMA was harvested from two sternal marrow spaces (35 ml each) into syringes containing preservative-free heparin (final concentration: 15 U heparin/ml BMA). The marrow aspirate was processed with a centrifuge to yield 6 ml of BMAC. Arthroscopically, BMAC and thrombin (10 : 1 volume ratio) were injected into the microfracture-treated defects using a syringe. The animals were confined to rest for two weeks, followed by increasing weight bearing and a second-look arthroscopy at 12 weeks. At sacrifice after 8 months, an MRI-based radiological evaluation revealed a significantly increased defect filling and improved integration with the surrounding cartilage in the BMAC-enhanced microfracture group compared with microfracture alone. BMAC treatment also yielded a significantly increased type II collagen and glycosaminoglycan contents with improved collagen fiber orientation of the repair tissue.

Saw et al. reported on the treatment of 15 young goats with full-thickness chondral defects (4 mm in diameter) in the intercondylar area of the knee treated with either subchondral drilling (9 drill holes per defect, 5 mm in depth, 0.6 mm in diameter), subchondral drilling with additional intra-articular injection of sodium hyaluronate (HA) (HA group), or subchondral drilling with intra-articular injection of both HA and BMAC (HA-BMAC group) [64]. The injections were administered on a weekly basis for 3 weeks, starting one week after surgery with defect creation and subchondral drilling. The BMA was harvested from the iliac crest and centrifuged to remove red blood cells and plasma and yielded a mean final volume of 4.4 ml BMAC. In the HA-BMAC group, each defect was injected with 400 *μ*l BMAC. Animals were mobilized without restrictions. At 6 months postoperatively, similar macroscopic findings were reported between drilling alone and the HA group. However, defects of the HA-BMAC group were almost completely filled and exhibited a smooth surface well in level with the adjacent normal cartilage. Histological evaluation revealed that BMAC/HA-enhanced marrow stimulation induced a significantly better histological grading of the articular cartilage repair with increased proteoglycan content and an improved lateral integration.

### 7.2. Clinical Evidence of BMAC-Enhanced Marrow Stimulation for Articular Cartilage Repair

Only four studies have been performed to investigate the specific clinical outcome of BMAC-enhanced marrow stimulation for chondral lesions. These were conducted in either the knee [71, 72] or the ankle joint [73, 74] (Table 2). Microfracture was the main marrow stimulation technique, applied in three studies [71–73], subchondral drilling only once [74]. In all the investigations, BMA was harvested from the iliac crest and processed with various commercially available centrifuge systems to generate BMAC.

De Girolamo et al. examined cellular characteristics and pain or adverse events in 11 patients with chondral lesions (Outerbridge types III or IV [75]) undergoing microfracture in combination with implantation of a type I/III porcine collagen matrix and application of BMAC [71]. Bone marrow from the iliac crest was harvested and centrifuged to obtain a concentrated phase containing mononuclear cells. Cellular characteristics were compared with samples obtained from the microfractured defect site. The authors reported that more cells with an MSC phenotype (CD34^−^/CD45^low^/CD271^high^) were found in the bone marrow from the iliac crest (0.04%) than from the subchondral location of the defect (0.02%). Clinically, no pain or adverse events were seen. The clinical outcome was not compared to a negative control group. Gobbi et al. treated 50 patients with chondral lesions of the knee (ICRS grade IV) with microfracture (microfracture group; median lesion size of 4.5 cm^2^) or a hyaluronan- (HA-) based scaffold plus BMAC (HA-BMAC group; median lesion size of 6.5 cm^2^) [72]. BMA was centrifuged to obtain a concentration of bone marrow cells approximately 6 times the baseline value. The hemotoxin batroxobin, also known as reptilase, was added to activate BMAC (from the iliac crest) to produce an adhesive clot. The clot was implanted into the cartilage defect that was prepared in a similar manner as for the microfracture group. It was covered with a HA-based scaffold and secured to the surrounding cartilage by sutures and/or fibrin glue. Weight bearing was restricted for the initial 4 weeks postoperatively. After 2 years, the HA-BMAC group obtained a normal or nearly normal International Knee Documentation Committee (IKDC) objective score in 100%, the microfracture group only in 64%. HA-BMAC-treated patients maintained a significantly improved knee function at 5 years according to Tegner and IKDC objective scores compared with microfracture-treated patients.

The concept of applying BMAC to improve cartilage repair has been also studied for defects of the talus [9]. Hannon et al. compared microfracture alone with BMAC-enhanced microfracture of talar defects in 34 patients [73]. Approximately 60 ml of BMA was extracted from the iliac crest and centrifuged to generate 3 ml of BMAC for each defect site. In the BMAC-enhanced microfracture group, BMAC was injected into the defect site under arthroscopic control after the subchondral plate had been penetrated with multiple microfractures. The Foot and Ankle Outcome Score (FAOS) pain score and the short form 12 (SF-12) general health questionnaire physical component summary (SF-12 PCS) score improved significantly from before to after surgery in both groups. Yet, the Magnetic Resonance Observation of Cartilage Repair Tissue (MOCART) score in the BMAC-enhanced microfracture group was significantly higher than that in microfracture alone group, indicating a significantly better morphological outcome compared with the microfracture alone group. MRI also revealed that BMAC-enhanced microfracture induced significantly less fissuring and fibrillation of the articular cartilage surface than microfracture treatment without adjunct. Lanham et al. evaluated 12 patients with full-thickness chondral defects of the talus (6-7 mm in depth) treated either with BMAC-enhanced subchondral drilling covered by a collagen scaffold or with particulated juvenile articular cartilage [74]. The collagen scaffold consisted of a hydrated matrix of bovine collagen and glycosaminoglycans. Approximately 60 ml of BMA was harvested from the iliac crest and centrifuged to obtain 6 ml of BMAC. After a mean follow-up of 2 years (range 12–42 months), the American Orthopaedic Foot and Ankle Surgeons (AOFAS) score as well as the Foot and Ankle Ability Measure (FAAM; activities of daily living subscale) showed a statistically significant improvement in favor of particulated juvenile articular cartilage compared with BMAC-enhanced marrow stimulation in these 12 patients. The SF-12 health status questionnaire [76] revealed no significant differences between both treatment groups.

## 8. Effects of Bone Marrow Aspirate-Enhanced Marrow Stimulation on Subchondral Bone Repair

Marrow stimulation affects not only articular cartilage but also subchondral bone repair. Relevant changes of the subchondral bone that have been observed include, for example, the upward migration of the subchondral bone plate, the formation of intralesional osteophytes and of subchondral bone cysts, and a generalized impairment of the osseous microarchitecture below the treated defects [77]. Of note, no study has been performed to date to the best of our knowledge to assess the effect of BMAC-enhanced marrow stimulation on the subchondral bone compartment compared with marrow stimulation alone. Given the abovementioned specifications and biological features of BMA and BMAC, such a biological adjunct may possibly have a beneficial impact not only on the articular cartilage but also on its supporting osseous bed.

## 9. Conclusions and Future Directions

In summary, there is good evidence from translational studies that BMAC-enhanced microfracture results in a significantly increased defect filling, better structural parameters of the repair tissue, and improved integration compared with microfracture alone. The study of Gobbi et al. shows after 2 years significant improvements in clinical scores of patients with relatively large chondral defects (median lesion size: 6.5 cm^2^) treated with BMAC that was additionally covered with a Hyaluronan scaffold compared to microfracture-treated patients [72].

Interestingly, no study investigated the effect of unconcentrated BMA for osteochondral repair in either translational or clinical settings so far. Such a treatment might constitute an interesting negative control and also shed more light on the possible effect of concentration on repair. Also, no standard technique or protocol for BMA harvesting exists. The commercially available systems including centrifuges do not provide equivalent cell numbers and concentrations. The ideal volume of required BMA or BMAC for the treatment of a specific defect volume remains to be determined. Standardizations of all of these factors are needed, as they may provide a more scientific way of evaluating possible effects, especially as there is a large interindividual variety in the number of produced and secreted growth factors by the many cells within the BMA. As marrow stimulation affects the entire osteochondral unit, further research is also mandatory to investigate possible effects of BMA on the important aspect of subchondral bone changes following cartilage repair procedures.

Although the structural [78] and functional [79] clinical results of marrow stimulation are usually good, the fibrocartilaginous repair tissue is inferior to the original hyaline cartilage. Possibly, application of novel insights to the problem of fibrosis with its excess deposition of an inferior fibrous tissue may show new avenues towards the goal of achieving true hyaline articular cartilage regeneration. For skeletal muscle, numerous approaches have been developed to inhibit the fibrotic cascade [80] that may also be studied in articular cartilage repair [81]. As discussed earlier, a combination of simultaneously blocking angiogenesis and fibrosis could also be investigated for its effects on cartilage repair [82].

Another future opportunity of enhancing the possible therapeutic potential of BMA is to increase the rate of MSC survival, leading to improved therapeutic functions. Little is known on the viability of migrated MSCs at the site of the cartilage defect, when exposed to biomechanical stresses and the synovial fluid. Possibly, strategies to regulate apoptotic signaling and enhance cell adhesion, such as hypoxic preconditioning [83] or application of growth or antiapoptotic factors by in situ genetic modifications [84, 85], may improve the in situ survival of the MSCs [86, 87].

Forthcoming translational and clinical studies will help to address the effect of BMAC-enhanced marrow stimulation of chondral defects more in detail.

## Figures and Tables

**Table 1:** Translational studies of BMAC-enhanced marrow stimulation for articular cartilage repair.

Animal model	Animal number	Defect location	Defect type	Defect size	Additional treatment	Nature of biomaterials	Source of bone marrow	Concentration performed?	Method of concentration	Volume per defect	Study groups	Follow-up	Evaluation methods	Major findings	Ref.
Horse	12	Trochlear ridge	Full thickness chondral	15 mm diameter	Microfracture	n.a.	Sternum	Yes	BMA harvested from two sternal marrow spaces (each 35 ml). Mixed with preservative- free heparin	n.a.	(1) Microfracture	8 months	(1) Cytological and flow cytometry analysis	(1) No adverse reactions	[65]
									BMA (60 ml) was centrifuged. Yield: 6 ml of BMAC		(2) Microfracture + BMAC		(2) MRI	(2) Improved cartilage repair in BMAC group	
													(3) Macroscopic scoring	(3) MRI: increased defect fill, improved lateral integration	
													(4) Histological scoring	(4) More type II collagen, improved collagen orientation, more glycosaminoglycans in BMAC group	

Goat	15	Intercondylar region of knee	Full-thickness chondral	4 mm diameter	Subchondral drilling	Hyalgan (HA)	Iliac crest	Yes	BMA was centrifuged (1900 rpm, 10 min., at 10°C). Mean yield: 4.4 ml (range, 4.0 to 6.0 ml) of BMAC	400 *μ*l	(1) Subchondral drilling	6 months	(1) Macroscopic observation	(1) Macroscopy: similar between empty defects and both HA groups. HA + BMA group: almost complete, smooth surface in level with adjacent cartilage	[64]
											(2) Subchondral drilling + i.a. HA		(2) Histological scoring	(2) Better histological score in HA + BMA group with more proteoglycan staining and improved lateral integration	
											(3) Subchondral drilling + i.a. HA+ BMA injection				

BMA: bone marrow aspirate; BMAC: bone marrow aspirate concentrate; HA: Hyalgan (sodium hyaluronate); i.a.: intra-articular; n.a.: not available; Ref.: reference.

**Table 2:** Clinical studies of BMAC-enhanced marrow stimulation for articular cartilage repair.

Patient number	Defect location(s)	Defect type	Defect size	Additional treatment	Nature of biomaterials	Source of bone marrow	Concentration performed?	Method of concentration	Aspirate amount/defect	Study group(s)	Follow-up	Evaluation methods	Major findings	Ref.
11	Femoral condyle, patella	1 or 2 chondral defects, Outerbridge types III or IV	2–8 cm^2^	Microfracture	Type I/III porcine collagen matrix	(1) Iliac crest	Yes	BMA (24 ml) centrifuged (15 min) to obtain a concentrated phase containing mononuclear cells.	n.a.	(1) Microfracture + collagen membrane + BMAC	6 months	(1) FACS analysis	(1) More cells with MSC phenotype obtained from iliac crest than microfracture site.	[71]
						(2) Microfracture site						(2) Culture of bone marrow samples from iliac crest and microfracture site	(2) Only MSCs from bone marrow could be long-term propagated and efficiently differentiated in vitro.	
												(3) Clinical evaluations: pain, adverse events	(3) No pain. No adverse events.	

50	Patella, medial femoral condyle	Chondral defects, ICRS grade IV	Median lesion sizes of 4.5 or 6.5 cm^2^	Microfracture	Hyaluronic acid-based scaffold	Iliac crest	Yes	BMA (60 ml) centrifuged. Yield: cellular concentration ~6× baseline value.	n.a.	(1) Hyaluronic acid-based scaffold + BMAC (HA-BMAC)	2 and 5 years	(1) MRI	(1) 100% normal or nearly normal IKDC objective score at 2 years in HA-BMAC (microfracture 64%).	[72]
								Batroxobin enzyme used to activate the BMAC.		(2) Microfracture		(2) IKDC objective and subjective score	(2) HA-BMAC group maintained improved knee function at 5 years according to Lysholm, Tegner, IKDC objective and subjective scores.	
												(4) KOOS	(3) Higher score for HA-BMAC group according to Tegner, IKDC objective, and KOOS scores.	
												(5) Lysholm		
												(6) Tegner		
34	Talus	Osteochondral defects	0.5–2.2 cm^2^	Microfracture	n.a.	Iliac crest	Yes	n.a.	3 ml	(1) Microfracture	2.8–8.3 years	(1) FAOS pain subscale	(1) FAOS and SF-12 PCS score significantly improved in microfracture group after a mean of 6.4 years and in the microfracture + BMAC group after a mean of 4 years.	[73]
										(2) Microfracture + BMAC		(2) SF-12 PCS	(2) MOCART score in microfracture + BMAC group significantly higher than in microfracture group after 2 years.	
												(3) MRI	(3) Per MRI less fissuring and fibrillation in the microfracture + BMAC group after 2 years.	

12	Talus	Full-thickness chondral	1.0–3.9 cm^2^	Particulated juvenile articular cartilage (PJAC); subchondral drilling	Bovine type I collagen and glycos-amino-glycan	Iliac crest	Yes	BMA (60 ml) centrifuged. Yield: 6 ml of BMAC	6 ml	(1) Subchondral drilling + BMAC + collagen scaffold	2.1 years (range: 1.0–3.5 years)	(1) AOFAS score	Better clinical outcome for the subchondral drilling + PJAC group according to higher AOFAS and FAAM scores after 2 years.	[74]
										(2) Subchondral drilling + PJAC		(2) FAAM score		
												(3) SF-12 score		

AOFAS: American Orthopaedic Foot and Ankle Surgeons; BMA: bone marrow aspirate; BMAC: bone marrow aspirate concentrate; FAAM: Foot and Ankle Ability Measure; FACS: fluorescence-activated cell sorting; HA: hyaluronic acid; ICRS: International Cartilage Repair Society; IKDC: International Knee Documentation Committee; KOOS: Knee Injury and Osteoarthritis Outcome score; Lysholm: Lysholm Knee Questionnaire; MOCART: Magnetic Resonance Observation of Cartilage Repair Tissue; MRI: magnetic resonance imaging; MSC: mesenchymal stem cell; n.a.: not available; PJAC: particulated juvenile articular cartilage; Ref.: reference; SF-12: short form 12 general health questionnaire; SF-12 PCS: SF-12 physical component summary score; Tegner: Tegner activity scale.
